# Implementation fidelity, student outcomes, and cost-effectiveness of train-the-trainer strategies for Masters-level therapists in urban schools: results from a cluster randomized trial

**DOI:** 10.1186/s13012-023-01333-9

**Published:** 2024-01-25

**Authors:** Ricardo Eiraldi, Gwendolyn M. Lawson, Henry A. Glick, Muniya S. Khanna, Rinad Beidas, Jessica Fishman, Quinn Rabenau-McDonnell, Tara Wilson, Rachel Comly, Billie S. Schwartz, Abbas F. Jawad

**Affiliations:** 1 Children’s Hospital of Philadelphia, Roberts Center for Pediatric Research, 2716 South Street, Room 8293, Philadelphia, PA 19146-2305 USA; 2 Department of Pediatrics, University of Pennsylvania Perelman School of Medicine, 3400 Civic Center Boulevard, Philadelphia, PA 19104 USA; 3 Department of Psychiatry, University of Pennsylvania Perelman School of Medicine, 3535 Market St, Philadelphia, PA 19104 USA; 4 Department of Biostatistics and Epidemiology, University of Pennsylvania, 423 Guardian Drive, Philadelphia, PA 19104 USA; 5 Department of Medicine, University of Pennsylvania Perelman School of Medicine, 3400 Civic Center Boulevard, Philadelphia, PA 19104 USA; 6 Wharton School, University of Pennsylvania, 3620 Locust Walk, Philadelphia, PA 19104 USA; 7 Leonard Davis Institute of Health Economics, University of Pennsylvania, 3641 Locus Walk # 210, Philadelphia, PA 19104 USA; 8 OCD and Anxiety Institute, 3138 Butler Pike # 200, Plymouth Meeting, PA 19462 USA; 9 Department of Medical Social Sciences, Northwestern University Feinberg School of Medicine, 625 North Michigan Avenue, Chicago, IL 60611 USA; 10 Message Effects Lab, Annenberg School for Communication, University of Pennsylvania, 3620 Walnut Street, Philadelphia, PA 19104 USA

**Keywords:** Train-the-trainer, Urban schools, Implementation, Consultation, Group cognitive behavioral therapy, Anxiety disorders

## Abstract

**Background:**

Little is known about the effectiveness and cost-effectiveness of train-the-trainer implementation strategies in supporting mental health evidence-based practices in schools, and about the optimal level of support needed for TT strategies.

**Methods:**

The current study is part of a larger type 2 hybrid cluster randomized controlled trial. It compares two train-the-trainer strategies, Train-the-Trainer (TT) and Train-the-Trainer plus ongoing consultation for trainers (TT +) on the delivery of a group cognitive behavioral treatment protocol for anxiety disorders. Participants were 33 therapists, 29 supervisors, and 125 students who were at risk for anxiety disorders from 22 urban schools. Implementation outcomes were implementation fidelity and treatment dosage. Student outcomes were child- and parent-reported symptoms of anxiety, child-reported symptoms of depression, and teacher-reported academic engagement. We estimated the cost of implementing the intervention in each condition and examined the probability that a support strategy for supervisors (TT vs TT +) is a good value for varying values of willingness to pay.

**Results:**

Therapists in the TT and TT + conditions obtained similarly high implementation fidelity and students in the conditions received similar treatment dosages. A mixed effects modeling approach for student outcomes revealed time effects for symptoms of anxiety and depression reported by students, and emotional disaffection reported by teachers. There were no condition or condition × times effects. For both conditions, the time effects indicated an improvement from pre-treatment to post-treatment in symptoms of anxiety and depression and academic emotional engagement. The average cost of therapist, supervisor, and consultant time required to implement the intervention in each condition was $1002 for TT and $1431 for TT + (*p* = 0.01). There was a greater than 80% chance that TT was a good value compared to TT + for all values of willingness to pay per one-point improvement in anxiety scores.

**Conclusions:**

A TT implementation approach consisting of a thorough initial training workshop for therapists and supervisors as well as ongoing supervision for therapists resulted in adequate levels of fidelity and student outcomes but at a lower cost, compared to the TT + condition that also included ongoing external expert consultation for supervisors.

**Trial registration:**

ClinicalTrials.gov identifier: NCT02651402.

**Supplementary Information:**

The online version contains supplementary material available at 10.1186/s13012-023-01333-9.

Contributions to the literature • Implementation fidelity and treatment dosage were similar between therapists in TT and TT + . Therapists in both conditions implemented the intervention with relatively high levels of fidelity.

• Participant students in TT and TT + showed similar levels of improvement in symptoms of anxiety, Depression, and emotional disaffection.

• TT appeared to be good value as it was found to be effective for obtaining adequate levels of fidelity and student outcomes at a lower cost, compared to TT + 

• This study advances the implementation science literature by demonstrating the amount and type of support provided within a TT implementation strategy that leads to acceptable levels of fidelity at a reasonable cost.

## Background

School districts in large urban centers typically do not have internal capacity to adequately meet student mental health needs. To address this, many school districts in the USA contract with mental health agencies to provide services in schools [1]. Therapists employed by these agencies typically have a Masters-level education and seldom receive adequate training in evidence-based practices (EBPs) [2]. Studies have shown that effective implementation models for community therapists include an initial training workshop followed by supervision [3–5]. Due to financial pressures, most school-based therapists, especially those funded by Medicaid, do not receive ongoing clinical supervision after participating in a training workshop [6, 7]. Agencies that provide services in urban schools typically do not have the internal capacity or financial resources to offer ongoing direct supervision or external consultation to their therapists [4, 7].

A potentially cost-effective implementation strategy is the Cascade or Train-the-Trainer (TT) approach, in which therapists or clinical supervisors are trained to implement EBPs and then they train other therapists [8]. The optimal level of implementation support for supervisors within a TT implementation strategy has not been determined. Understanding the optimal level of implementation support within a TT implementation strategy in the urban school context may be generalized to inform the use of TT implementation strategies in other resource-constrained contexts.

### Train-the-trainer effectiveness outcomes

A literature review using TT to train practitioners in a variety of areas (e.g., physical injuries, health and mental health problems, HIV/AIDS care) revealed better patient outcomes compared to training as usual [9]. School-based studies using TT have reported improved nutrition knowledge among third-grade students using a curriculum delivered by school personnel [10] and increased knowledge of sexual abuse among fifth-grade students with an intervention implemented by school staff [11]. A study employing individual therapy with children with anxiety, depression, trauma, or conduct problems showed that a TT approach used by outpatient therapists was effective in improving and sustaining child clinical outcomes [12].

### Train-the-trainer implementation outcomes

Emerging evidence suggests that TT strategies show promise in improving therapist implementation outcomes, including competence [4, 13], knowledge [9], penetration [14], and fidelity [12]. For example, in one study, a TT approach with community therapists was effective at increasing and sustaining therapist fidelity [12], and, in another study, TT was associated with higher penetration of Parent–Child Interaction Therapy (PCIT) compared to a learning collaborative condition and a distance education condition [14]. In a study conducted in a school setting, school teams trained via a TT approach to assist students with academic and behavioral difficulties using a problem-solving approach, scored higher on several indexes of team effectiveness (e.g., communicating clearly with one another; developing manageable interventions for teachers and students) compared to teams in control schools [15].

The effects of TT have been investigated more often for adherence or content fidelity (i.e., extent to which the prescribed components of the intervention are implemented), than for dosage (i.e., number of sessions delivering training material), or therapeutic competence or process fidelity (i.e., therapist’s skill and judgment in delivering intervention components) [16, 17].

In summary, previous studies have shown that TT is likely an effective implementation strategy for improving patient and child outcomes. However, none of these studies examined the effectiveness of TT implementation strategies for child mental health outcomes in the school setting, or examined all three components of fidelity.

### Train-the-trainer cost-effectiveness

Very few studies have been conducted examining the cost and cost-effectiveness of TT in schools [18–21]. In general, TT is seen as being more cost-effective than training by expert trainers because the initial costs of preparing therapists as trainers are often offset by lower clinician training and supervision costs for those trained by the new trainers [19]. However, studies have not resulted in consistent findings in this area [21, 22].

Regarding the cost of TT in relationship to student outcomes, a study analyzed the cost-effectiveness of an early childhood self-regulation intervention, comparing three different models of implementation across stages of intervention development: (a) trained research assistants (RAs) directly delivered the intervention to children; (b) RAs trained trainers, who then trained teachers to implement the intervention with students (i.e., TT); and (c) program faculty trained teachers to deliver the intervention to students [23]. Results showed that TT was the most cost-effective strategy for improving student self-regulation and that this training strategy remained the optimal strategy in sensitivity analysis.

Currently, the cost of TT in urban schools, and the cost-effectiveness of training trainers in relation to student outcomes, are unknown. Given the increased necessity to demonstrate the benefits of using EBPs vis-à-vis costs, agencies could benefit from studies examining the cost-effectiveness of improving student outcomes [18].

### Type of support provided within train-the-trainer

The effectiveness of TT could vary as a function of how the training is delivered (e.g., computer-aided, face-to-face, virtual) and what is included in the training (e.g., initial training workshop, additional ongoing consultation) [9]. The literature shows that therapists need ongoing supervision following a one-time workshop [24], and that interactive, multifaceted training works best [4, 9]. In a TT model within the context of schools with agency-employed mental health providers, this ongoing supervision could be provided by an agency therapist who is trained on EBPs and who can subsequently function as a supervisor. We do not yet know what type of implementation support works best for these supervisors. A TT training strategy for supervisors that only involves participation in a supervision training workshop would likely be more time-efficient and less expensive but might be linked to less favorable student and implementation outcomes, compared to a strategy that also provides consultation after an initial training workshop. It is important to know whether a one-time training for the supervisor is sufficient, or whether ongoing expert consultation is needed instead. Understanding this can help us design TT implementation strategies that are both effective and cost-effective.

### Current study

This study is part of a larger study aimed at evaluating the effectiveness of two group cognitive behavioral therapy (CBT) for anxiety (Friends of Life [FRIENDS] [25], and CBT Anxiety Treatment in Schools [CATS]) [26], and two implementation strategies (traditional Train-the-Trainer [TT], and Train-the-Trainer plus ongoing remote online consultation for supervisors [TT +]), using a three-arm, parallel group, type 2 hybrid effectiveness-implementation trial in 36 urban schools. For the current study, we compared implementation outcomes of TT vs. TT + and clinical effectiveness of the 8-session CATS manualized protocol under the two types of implementation support based on a sample of 22 schools (i.e., those receiving CATS with TT implementation support and those receiving CATS with TT + implementation support). Results pertaining to the effectiveness of FRIENDS vs. CATS using the TT strategy were presented elsewhere [27].

### Specific aims and hypotheses

The aims of the study were to assess therapists’ implementation outcomes (i.e., content fidelity and process fidelity to the group intervention; dosage), and pre-to-post- changes in student outcomes (i.e., student symptom severity of anxiety and depression, and teacher-reported academic engagement). We also aimed to estimate the cost and cost-effectiveness of the two implementation strategies. The study was originally designed to test the hypotheses that students in TT + would demonstrate larger decreases in symptoms of anxiety and depression, and larger increases in academic engagement from pre-to-post- compared to TT, and that TT + would yield higher content fidelity, process fidelity, and dosage scores compared to TT. We also expected that TT would still lead to acceptable levels of therapist fidelity (content fidelity ≥ 80%; process fidelity ≥ 4 on a 1–5 scale, 1 = not at all, 5 = very often). Finally, we expected that TT + would cost more than TT but TT would increase student outcomes sufficiently to make this training approach a good value. However, the study took place during the COVID-19 pandemic, which led to interruptions in participant recruitment, and is therefore underpowered to test these hypotheses. The study helps to better understand the optimal design of TT implementation strategies in under-resourced schools.

## Methods

### Study design and timeline

The manuscript follows the CHEERS and CONSORT reporting guidelines. Data for the type 2 hybrid [28] cluster randomized controlled trial with parallel groups of two TT implementation strategies [29] were collected between 2016 and 2020. We stopped delivering the interventions to students in March 2020. We collected data on groups that had covered 50% of the intervention content up to the time when on-site classes were suspended because of COVID-19. Data were not collected after March 2020; data post this period is considered missing. The methods were changed after commencement of the trial. The use of a semi-structured interview for student participant inclusion was no longer required because of participant burden (i.e., the interview was too lengthy).

### Participant inclusion criteria

- Any supervisor or therapist with a Master’s degree or higher, who was providing services in one of the participating schools, and was willing to be trained and who agreed to participate.
- Any student in grades 4–8, already enrolled in the mental health program at their school who was at or above the anxiety symptom severity cut-off (i.e., Total score ≥ 25 and/or above threshold on some of the subscale scores of the Screen for Child Anxiety Related Disorders (SCARED) [30] completed by a caregiver or the student).

Parental consent and student assent were required for participation. Students were required to show elevated anxiety symptoms but were not required to meet diagnostic criteria for any anxiety disorder. Our goal was to focus recruitment among students with moderate symptom severity levels of anxiety, who would be amenable to receiving treatment for anxiety via group therapy instead of individual therapy.

### Exclusion criteria

- Students with the classification of “Intellectual Disability” according to school records.
- Students who had diagnoses that would make participation in the study clinically inappropriate (i.e., psychotic or autism spectrum disorders, based on school records) because they would be unlikely to benefit from group CBT [31] or who presented at an acute risk to themselves or others, were excluded.

The schools where therapists, supervisors, and students participated were located within several low-income neighborhoods in a large city in the Northeast USA.

### Setting

#### Agency and school recruitment

To identify potential mental health agencies that provide prevention and treatment services in urban public and charter schools, we collaborated with the nonprofit organization that manages Medicaid funds and contracts with agencies to provide mental health services. We contacted 15 agencies via email and telephone, 10 agencies accepted our offer to conduct a brief presentation about the project, and 9 agencies agreed to be part of the study. The presentations, which were conducted in person or via video, included an overview of the study, an explanation of the randomization procedure of schools to condition, the voluntary nature of participation, and the potential benefits to students, schools, agencies, supervisors, and therapists. Agency administrators were told that an important goal of the study was to help create internal capacity within the agencies to provide quality supervision for the implementation of EBPs for anxiety disorders in children. After agreeing to participate, the administrators provided a list of schools that they thought would be good candidates for participation. Following the initial school selection, investigators met with school administrators to provide an in-depth description of the project and ask if they would agree to participate. Thirty-six schools that had a service contract with the 9 agencies to provide services in the schools agreed to participate in the larger study. Data for the present study originate from 26 schools receiving CATS [29].

### Interventions

#### CBT treatment protocol

The intervention implemented by school therapists was CBT for Anxiety Treatment in Schools (CATS) [26]. CATS is a manualized group-based program based on the evidence-based principles of cognitive-behavioral therapy (CBT) for anxiety in children and adolescents. The components of the treatment are based on the *Coping Cat* [32] and FRIENDS for Life [25] manualized treatment protocols. It teaches children how to recognize feelings of and physical reactions to anxiety, clarify thoughts and feelings in anxiety-provoking situations, develop a coping plan, evaluate their own performance, and practice self-reinforcement (see Table 1). A more thorough description and effectiveness of CATS can be found elsewhere [25].

**Table 1:** Treatment protocol

CBT for anxiety treatment in schools [26]
Session 1: Welcome and introduction to feelings
Session 2: Physiological symptoms of anxiety and relaxation training
Session 3: Identifying my feelings and thoughts
Session 4: Choose how I want to think and feel
Session 5: Actions that will get me closer to my goal
Session 6: Now try it!
Session 7: Remember, I can!
Session 8: Review, practice, and party!

#### Implementation strategies

The study compared two implementation strategies: (a) TT (i.e., initial training for therapists and supervisors), and (b) TT + (i.e., initial training for therapists and supervisors plus ongoing weekly remote online consultation for supervisors). Therapists in both implementation strategies implemented CBT for Anxiety Treatment in Schools (CATS) [26] with participant students.

#### Training and consultation

The initial training workshop for supervisors and therapists in TT and TT + was conducted in the schools as a one-time session or split over several days, depending on need. At the conclusion of the training, supervisors and therapists were administered a test to assess knowledge of the concepts of the anxiety treatment protocol [33]. Participants who scored below 80% were provided further training in the areas in which they scored low. Subsequent supervision of therapists by supervisors took place in the schools. Consultation for agency supervisors (on how to be effective supervisors) was conducted remotely via the Zoom platform. Research Electronic Data Capture (REDCap) and ShareFile, a secure data-sharing platform, were used to enable the uploading and remote watching of treatment video sessions and therapist-to-supervisor supervision sessions.

All activities related to the training and consultation of supervisors and supervision of therapists were organized around the Interactive System Framework (ISF) [34]. The ISF is composed of three interrelated systems: The Synthesis and Translation System (STS), the Support System (SS), and the Delivery System (DS). The function of the STS is to distill information innovations and prepare them for implementation by service providers. The SS supports the work of those who put the innovation into practice, and the DS implements the innovations in “real world” settings [34]. We used the SS to organize the support for supervisors and therapists and the DS to organize treatment delivery by therapists. A more detailed description of project activities within the ISF is provided in the study protocol paper [29] and Supplementary information 1.

#### Training and consultation for supervisors

The goal of the training for supervisors was to train in supervision best practices [35], as well as on CBT principles, best practices for managing groups, and content and procedures for the CATS treatment manual. We adapted a training approach we have used in prior work [36, 37] to train supervisors. The training included an 8-h initial training workshop for supervisors in both conditions. The initial training was sometimes split into several sessions to accommodate the schedule of busy clinicians. When the group intervention did not begin within a month of supervisor training, supervisors in both conditions also received a one-time, one-hour booster training session. Supervisors in TT + subsequently received ten 45-min weekly consultation sessions. The training and consultation of supervisors (see Table 2 and Supplementary information 1) were provided by expert-supervised research consultants.

**Table 2:** Support provided to supervisors and clinicians

Support provided to supervisors by condition	
Train-the-Trainer (TT)	Train-the-Trainer-Plus (TT +)
- 8-h initial training on EBPs followed by booster training in subsequent project years	- 8-h initial training on EBPs followed by booster training on subsequent project years
- Training about clinical supervision	- Training about clinical supervision
- Knowledge test	- Knowledge test
	- Ten weekly 60-min consultation sessions in the first year, and 5 60-min consultation sessions for returning supervisors via Zoom platform
Support provided to clinicians for both conditions	
Provided by research team	Provided by Agency Supervisors
- Four days of initial training	- Eight supervision sessions: Session preparation; self-reflection; goal setting; content and process fidelity feedback
- Video recordings of well-executed treatment main components	- Video recordings of well-executed treatment main components

#### Training of therapists

Therapists in both conditions participated in an initial 8-h training workshop conducted by research consultants. The initial training was sometimes split into several sessions, as needed. Supervisors were expected to provide 9 supervision sessions (60 min each) to therapists. The supervision sessions were divided into group preparation (e.g., preparing for upcoming session) and coaching (e.g., performance feedback).

#### Training of consultants

Consultants (two post-doctoral psychology fellows and a Masters-level clinician in counseling psychology) were trained in a three-step process. (1) In advance of their training, they were provided the treatment manual and child workbook to begin reading for an overview of the protocol. (2) They participated in a 3-h workshop conducted by a licensed psychologist who is an expert trainer on CBT for anxiety. The training included a didactic presentation, video examples, live modeling by the expert, and role-plays. (3) Following the initial training workshop, the psychologist provided 10 biweekly 60-min supervision sessions. Prior to supervision sessions, the psychologist watched a video of consultation sessions conducted by the consultant. Supervision focused on ensuring that consultants (a) accurately and consistently communicated CBT principles; (b) encouraged and positively reinforced supervisors for their efforts; and (c) delivered didactics consistently across groups. The consultants participated in annual “refresher” trainings in subsequent years.

### Outcome measures

We measured implementation fidelity (content and process) and treatment dosage; pre- to post-changes in anxiety symptom severity and academic engagement. Measurement data were provided by independent coders, therapists, supervisors, research consultants, parents, students, and teachers. Table 3 presents the primary outcome instruments with information about their psychometric properties. The measures are organized by construct, time point, informant, and method.

**Table 3:** Measurement instruments presented by construct, timepoint, method, and informant

Construct	Instrument	Instrument characteristics	Timepoints	Method	Informant
Training					
Knowledge of CBT and treatment of anxiety	Adapted Knowledge Test (KT) [33]	The adapted version of the KT measures knowledge of CBT treatment for anxiety based on 8 multiple-choice questions with 4 possible response options, and two true/false questions. Possible scores range from 0 to 10. Alternate paper forms were used for repeated measurement	Initial training	Questionnaire	Therapist
Sample description					
Family characteristics	Demographic information	Age, grade, gender, race/ethnicity, and socioeconomic status were administered via REDCap	Pre-diagnostic evaluation	Questionnaire	Parents
Screening					
Anxiety disorders	Screen for Child Anxiety Related Disorders (SCARED) [30]	41-item, 3-point scale (0 = not true or hardly ever true to 2 = very true or often true) paper version organized around five scales and a Total Score. The SCARED has excellent psychometric properties and has been used in community settings as a screening instrument for anxiety disorders [30]	Pre-diagnostic evaluation	Rating scale	Parents and teachers
Content fidelity	Content Fidelity Checklist [38]	Raters use a yes/no response scale to indicate whether the implementer covered a particular component. In a previous study we obtained good inter-observer agreement (.812, range .671–.944) [36]. We used the total % score for the analysis	Ongoing	Video coding	Independent coding of therapist behavior
Process fidelity	Process Fidelity Checklist [36]	Ten items are rated on a scale of 1 to 5, with 1 being *Not at all* and 5 being *Very Often*. A coding manual provides operational definitions for each item and were used to train coders. On a previous study [36], we conducted an exploratory factor analysis (EFA). The EFA yielded two factors. Factor 1 (*Active engagement*) and Factor 2 (*Organized teaching*) accounted for 39% and 38% of the variance respectively. The overall Cronbach alpha for the total score as well as for factor 1 and factor 2 were excellent and equal to .92, .93, and .90 respectively. We used the average *Total* and factor scores for analysis for the present study	Ongoing	Video coding	Independent coding of therapist behavior
Dosage	Dosage Report	Number of sessions from the manual administered to students was reported by therapists and certified by supervisors using a form completed via REDCap	Ongoing	Questionnaire	Therapists / Supervisors
Anxiety symptoms	Multidimensional Anxiety Scale for Children–2nd Edition (MASC-2) [39]	The MASC-2 is a 50-item, 4-point rating scale (0 = never to 3 = often) paper instrument organized around six subscales and a Total score used for the assessment of anxiety symptoms in children. The instrument has strong psychometric properties and it is effective for measuring treatment effects [39]. The current sample Cronbach Alpha scores were .92 for the parent version, and .91 for the child version. We used the Total score in analyses	Pre- and post-treatment	Rating scale	Parents and students
Depression symptoms	Children’s Depression Inventory–2nd Edition (CDI-2) [40]	The CDI-2 is a 28-item, 3-point rating scale instrument organized around six subscales and a Total score used for the assessment of depression symptoms in children. The instrument has strong psychometric properties and it is effective for measuring treatment effects [40, 41]. We used the Total score in analyses. The Cronbach Alpha score was .77	Pre- and post- treatment	Rating scale	Students
Student academic engagement	Engagement versus Disaffection with Learning-Teacher Report (EvsD-Teacher) [42, 43]	The paper version of the EvsD was completed by teachers for all students in the study. This is a 20-item, four-point (1 = not at all true; 4 = very true) instrument with four sub-scales: (a) *Behavioral Engagement (BE)*; (b) *Emotional Engagement (EE)*; (c) *Behavioral Disaffection (BD);* and (d) *Emotional Disaffection (ED)*. Scores in the BD and ED are reverse scored, so that higher scores indicate better engagement across all four subscales. Reported internal consistency for students in grades 3–6 was .81–.87 for the four subscales [42, 43]. The internal consistency of the subscales in the full baseline sample were BE *α* = .89; EE *α* = .90; BD *α* = .76; and ED *α* = .73. We used the average score for each of the four scales at pre- and post-participation in treatment	Pre- and post-treatment	Rating scale	Teachers
Cost	Administrative records	Information was collected via REDCap about therapist time, agency supervisor time, and expert supervision time implementing the program	Ongoing	Time report	Therapists and supervisors

Data were collected on-site at the schools, via REDCap, and over the phone. The cost assessment measures included training time for therapists and agency supervisors, as well as for those providing the training; time subsequently spent in the supervision of therapists by agency supervisors; time of outside consultants consulting with supervisors; time spent conducting group sessions with students; as well as assessment and documentation time. Times were derived from administrative records of session times and student attendance, and diaries (completed during 4 1-week periods throughout the trial). Therapist, supervisors, and expert consultant time was costed out using data on average salary and benefits for those in job categories matching the staff providing these services. Hourly wages were derived based on an assumption of a 1920 (48 weeks × 40 h) hour work year. Wages were assigned to therapists and supervisors based on their years of experience.

### Independent coder training and reliability monitoring

Group treatment and consultation sessions in both conditions were video-recorded. Video recordings of group treatment sessions were coded by an independent coder for content and process fidelity. Coders, Masters-level research assistants who had been trained to a standard of reliability established by a licensed psychologist, were responsible for coding all student treatment video sessions. Two coders were trained to a reliability standard as follows: coders read the coding manual, including item definitions, exemplars, and descriptions of differentiation from other items. Then, together with the trainer, the coders observed four representative videos, reviewed specific session segments, and practiced scoring sessions. The coders then rated four new video recordings. Coders were approved for coding after their ratings achieved acceptable interrater reliability at the individual item level (content fidelity: Kappa ≥ 0.80 and process fidelity: ICC [2, 2] > 0.80). Once coding commenced, videos were randomly assigned to coders. Monthly meetings were held with the coding team to discuss new videos in order to prevent drift. A total of 302 student sessions were conducted (CATS TT, *n* = 121 student sessions; CATS TT + , *n* = 181 student sessions), and 286 (95%) sessions were video-recorded (CATS TT, *n* = 117; CATS TT + , *n* = 169). The first coder was randomly assigned 178 (62%) sessions for content and process fidelity. Of 178 sessions, 56 sessions (31%) were randomly selected for double coding by a second rater to evaluate inter-rater reliability. The ICC (2,2) was 0.70 for content fidelity (i.e., total percent of content covered), 0.67 for the Active Engagement process fidelity score, and 0.65 for the Organized Teaching process fidelity score.

### Sample

**Fig. 1:**
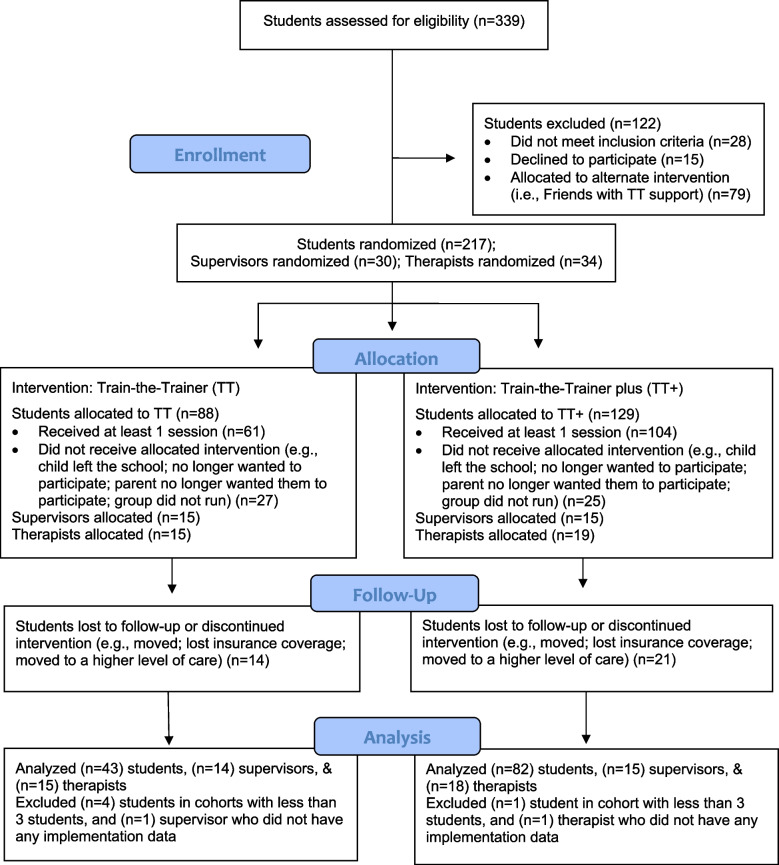
Consort flow diagram for the cluster-randomized study

Information about supervisors, therapists, and students who were eligible for the study, who consented, and whose schools were randomly assigned to CATS with TT and CATS with TT + , is shown in CONSORT Fig. 1.

Eighty-eight students, 15 supervisors, and 15 therapists were allocated to TT and 129 students, 15 supervisors, and 19 therapists were allocated to TT + . Fourteen students in TT and 21 students in TT + were lost to follow-up. No supervisors or therapists were lost to follow-up.

### Randomization

New schools were recruited every year starting in 2016 (until 2019), and stratified by school size (≥ 650 students or < 650 students). For the larger study, schools were randomized to receive one of the three conditions in 1:1 ratio using a computerized random assignment program generated using the SAS software [44]. A total of 22 schools were randomized to CATS with TT or CATS with TT + , and students received at least one session of therapy. Schools participated in the study for a maximum of 2 years. Agency supervisors and therapists participated in the conditions assigned to the school. Data collection was originally scheduled to end in 2021 but it was suspended in March 2020 due to the COVID-19 pandemic.

### Data analytic plan

We compared baseline demographic information (e.g., age, ethnicity, race, gender), student outcomes measured at baseline using the two samples independent t-test or the non-parametric Wilcoxon rank sum test for comparing continuous variables, and Fisher’s exact test or the chi-square test for comparing frequencies of baseline demographic measures across the two conditions. For implementation outcomes, we compared differences in mean fidelity (content and process fidelity) and dosage between the two conditions using the independent two-sample *t*-test or the non-parametric Wilcoxon rank sums test.

We used a mixed-effects modeling approach to compare student outcomes between TT and TT + . These models include both fixed effects (representing the overall treatment effects) and random effects (capturing the cluster-specific variation). The random effects component accounts for the clustering within schools by assuming that the treatment effects can vary across clusters. We assumed that the intercepts and slopes for each student were random effects, treatment conditions and time (pre-/post-) were the fixed effects, and students were nested (cluster) within their randomized school. The covariance structures related to the mixed-effects models were defined as unstructured (UN). The reported results were based on the entire distribution of fixed and random effects. We compared differences in mean fidelity (content and process fidelity) between the two conditions using the two samples independent *t*-test and the non-parametric Wilcoxon rank sums test, when appropriate.

The full analysis set (FAS) was defined to include all participants (*n* = 125) who (a) were randomized into treatment condition; (b) were in a school with at least 3 students receiving therapy; (c) received at least one session of therapy; (d) had post-baseline assessment data. The FAS consisted of 43 students who received group intervention under TT and 82 students who received intervention under TT + (see Fig. 1).

For the economic analysis, we estimated the cost per 1-point improvement in each cohort’s average child self-report MASC-2 score from a payer’s perspective. An average 1-point improvement for all students in a cohort, not for a single child in the cohort. The numerator of the incremental cost-effectiveness ratio was the difference in average costs per cohort between the 2 groups (i.e., TT + minus TT). Positive values for the numerator indicate that TT + increased costs compared to TT. The denominator was the difference in average change in MASC-2 scores (i.e., TT + average minus TT average changes). We refer to a 1-point reduction in MASC-2 scores as a 1-point improvement. In addition, we plot the empirical joint distribution of the differences in costs and effects on the cost-effectiveness plane and use it to depict the 95% confidence interval for the cost-effectiveness ratio. We also plotted the cost-effectiveness acceptability curve, which reports the probability that a support strategy for supervisors (TT vs TT +) is good value for varying values of willingness to pay for a 1-point improvement in MASC-2 scores. The time horizon for each observation was the duration of a cohort (approximately 8 weeks), and thus there was no discounting. Costs are reported in 2022 U.S. dollars (i.e., the source of the wage data). Sampling uncertainty was assessed by bootstrapping the trial data.

## Results

### Therapist and supervisor characteristics at baseline

Twenty-nine supervisors (14 in TT, 15 in TT + ; 79% females), and 33 therapists (15 in TT, 18 in TT + ; 79% females) participated in the study. Most supervisors (61%) and therapists (59%) self-identified as Black/African American. The highest education attainment was a Masters degree for 94% of supervisors and 100% of therapists. Most supervisors (72%) and therapists (76%) had provided mental health services in schools for less than 10 years. Therapists and supervisors did not differ on any of the examined characteristics (see Table 4).

**Table 4:** Demographic characteristics of therapists and supervisors

Characteristic	Train-the-Trainer	Train-the-Trainer +	Statistical test	*P* value
Supervisors (*N* = 29)				
Gender				
Male	3 (23.08)	3 (18.75)	Fisher’s exact	1.0
Female	10 (76.92)	13 (81.25)		
Age	42.85 (10.06)	41.06 (10.99)	Pr >|t|	0.66
Ethnicity				
Hispanic	1 (7.69)	0 (0.00)	Fisher’s exact	0.44
Not Hispanic	12 (92.31)	16 (100.00)		
Race (*n* = 28)				
White	4 (33.33)	7 (43.75)	Fisher’s exact	0.70
Black/African American	8 (66.67)	9 (56.25)		
Highest academic degree				
Doctorate	0 (0.00)	1 (6.25)	Fisher’s exact	1.0
Master’s	13 (100.00)	15 (93.75)		
Years providing clinical supervision	6.69 (6.14)	6.75 (7.46)	Pr >|t|	0.98
Therapists (*n* = 33)				
Gender (*N* = 33)				
Male	5 (33.33)	2 (11.11)	Fisher’s exact	0.20
Female	10 (66.67)	16 (88.89)		
Age	37.00 (7.09)	38.56 (8.00)	Pr >|t|	0.56
Ethnicity				
Hispanic	0 (0.00)	1 (5.56)	Fisher’s exact	1.0
Not Hispanic	15 (100.00)	17 (94.44)		
Race (*n* = 32)				
White	6 (42.86)	7 (38.89)	Fisher’s exact	1.0
Black/African American	8 (57.14)	11 (61.11)		
Highest academic degree				
Master’s	15 (100.00)	18 (100.00)		
Years of experience as a therapist in the school setting (*n* = 30)	3.77 (3.14)	3.06 (2.73)	Pr >|t|	0.51

### Student characteristics at baseline

Most students were male (75%), African American (62%), and attended fourth (typically age 9; 40%), fifth (age 10; 27%), or sixth (age 11; 18%) grade. Participant age in TT was 10.91 (SD = 1.49) years, and 10.85 (SD = 1.45) years in TT + (*p* = 0.85). One-hundred percent of students were eligible for subsidized lunch. There were no statistically significant differences between conditions on gender, race, ethnicity, or grade. See Table 5.

**Table 5:** Demographic characteristics of students allocated to condition at baseline and received at least one treatment session

Characteristic	Train-the-Trainer *N* SD/(%)	Train-the-Trainer + *N SD/*(%)	Statistical test	*P* value
Age (*n* = 125)	10.91 (1.49)	10.85 (1.45)	Ttest_Pr >|t|	0.45
Gender (*n* = 125)				
Male	29 (67.44)	65 (79.27)	Fisher’s exact	0.19
Ethnicity (*n* = 120)				
Hispanic or Latino	14 (11.67)	31 (25.83)	Fisher’s exact	0.84
Not Hispanic or Latino	26 (21.67)	49 (40.83)		
Race (*n* = 116)				
Black/African American	24 (20.69)	48 (41.38)	Chi-square	0.39
White	8 (6.90)	11 (9.48)		
Asian/Native Hawaiian	1 (.64)	1 (.64)		
More than one race	5 (4.31)	5 (4.31)		
Not reported	3 (2.59)	12 (10.34)		
Grade (*n* = 125)				
4th	18 (41.86)	32 (39.02)	Chi-square	0.97
5th	11 (25.58)	23 (28.05)		
6th	7 (16.28)	16 (19.51)		
7th	4 (9.30)	7 (8.54)		
8th	3 (6.98)	4 (4.88)		
Child, parent, teacher rating scales				
MASC 2 child (*n* = 125)	58.56 (12.54)	54.51 (10.87)	Ttest_Pr >|t|	0.06
CDI 2 child (*n* = 119)	59.40 (12.08)	59.19 (10.69)	Ttest_Pr >|t|	0.93
MASC 2 parent (*n* = 91)	64.16 (16.16)	57.88 (15.39)	Ttest_Pr >|t|	0.07
EvsD behavioral engagement (*n* = 122)	16.93 (3.11)	16.31 (3.08)	Ttest_Pr >|t|	0.30
EvsD emotional engagement (*n* = 122)	15.10 (3.61)	15.10 (3.63)	Ttest_Pr >|t|	0.99
EvsD behavioral disaffection (*n* = 122)	11.95 (3.70)	12.20 (3.20)	Ttest_Pr >|t|	0.70
EvsD emotional disaffection (*n* = 122)	12.90 (3.51)	13.55 (3.25)	Ttest_Pr >|t|	0.31

### Sample for cost-effectiveness analyses

The sample for the cost-effectiveness analyses was assembled at the level of group cohorts (i.e., the supervisor, therapist, and students who were assigned to TT or TT +). Some supervisors and some therapists led more than one cohort; however, no students participated in more than one cohort. Inclusion in the analysis required at least one supervisor or therapist time contribute one change in student MASC-2 score per cohort. Data were available from 35 cohorts. Due to supervisors participating in multiple cohorts, 28 supervisors contributed time data for 64 weeks. Twenty-eight therapists contributed time data for 68 weeks. One hundred nine students contributed a change in MASC-2 score, with at least one in each of the 35 cohorts.

### Implementation outcomes

There were no group differences for content fidelity (TT = 0.89 [SD = 0.12]; TT +  = 0.94 [SD = 0.08]), Wilcoxon rank sum test = 1.49, *p* = 0.14. There were no group differences for process fidelity, for Active Engagement (TT = 3.88 [*SD* = 0.27]; TT + 4.00 [SD = 0.20]), *t* = 1.60*, p* = 0.12, and Organized Teaching (TT = 3.33 [SD = 0.53]; TT +  = 3.54 [SD = 0.50]), *t* = 1.23, *p* = 0.23). Also, there were no group differences for session dosage (TT, *N* = 44, 6.32 [SD = 0.08]; TT + , *N* = 83, 5.76 [SD = 2.17]), Wilcoxon rank sum *Z* test = 1.66, *p* = 0.10.

### Student outcomes

For student report measures, there was an effect of time (*F* = 8.42, *p* = 0.004) for MASC-2 total scores, where student-reported MASC-2 scores were lower in both conditions at post-treatment compared to baseline. The condition × time interaction was not significant (*F* = 1.62, *p* = 0.20). There was a time effect (*F* = 8.68, *p* = 0.004) for the CDI-2, where the student-reported CDI-2 was lower at post-treatment compared to baseline. The condition × time interaction was not significant (*F* = 0.68, *p* = 0.41). For the parent-reported MASC-2 scores, the effect of time was not significant (*F* = 0.56, *p* = 0.45), and the condition × time was also not significant (*F* = 0.37, *p* = 0.55). For the teacher reports, there was a time effect (*F* = 7.41, *p* = 0.007) for the Emotional Disaffection subscale of the EvsD questionnaire, where the post-treatment scores were higher at post-treatment (reflecting better engagement) compared to baseline for both conditions. The treatment × condition interaction for this subscale was not significant (*F* = 0.07, *p* = 0.79). The time effect was not significant for any of the three other EvsD subscales (all *p*’s > 0.16). None of the condition × time interactions were significant (all *p*’s > 0.53); see Table 6.

**Table 6:** Mixed effects for student, parent, and teacher rating scales

Outcome measure	TT condition		TT + Condition		Condition		Time		Condition * Time	
	Pre *M*(*SE*)	Post *M*(*SE*)	Pre *M*(*SE*)	Post *M*(*SE*)	Estimate (SE)	*P *value	Estimate (SE)	*P *value	Estimate (SE)	*P* value
Student report										
MASC-2	56.44 (0.99)	53.19 (0.99)	55.80 (0.76)	54.54 (0.76)	1.35 (1.26)	0.77	3.26 (1.26)	0.00	− 1.99 (1.56)	0.20
CDI-2	59.44 (0.81)	57.96 (0.81)	59.39 (0.57)	56.77 (0.57)	− 1.20 (0.99)	0.39	1.47 (1.14)	0.00	1.15 (1.39)	0.41
Parent report										
MASC-2	60.02 (2.03)	57.79 (2.03)	59.25 (1.19)	59.01 (1.19)	1.24 (2.36)	0.62	2.25 (2.86)	0.46	− 2.01 (3.32)	0.57
Teacher report										
EvsD–behavioral engagement	16.60 (0.26)	16.37 (0.26)	16.48 (0.19)	16.54 (0.19)	0.17 (0.32)	0.89	0.23 (0.37)	0.45	− 0.29 (0.46)	0.55
EvsD–emotional engagement	15.04 (0.35)	14.69 (0.35)	15.07 (0.25)	14.99 (0.25)	0.30 (0.43)	0.48	0.35 (0.50)	0.67	− 0.81 (0.61)	0.70
EvsD–behavioral disaffection	12.05 (0.30)	12.45 (0.30)	12.11 (0.21)	12.45 (0.21)	0.00 (0.37)	0.90	− 0.40 (0.43)	0.16	0.06 (0.52)	0.90
EvsD–emotional disaffection	13.23 (0.33)	14.08 (0.33)	13.40 (0.23)	14.08 (0.23)	0.01 (0.40)	0.76	− 0.85 (0.46)	0.01	0.15 (0.57)	0.79

### Cost and cost-effectiveness

On average therapists spent 1.9 h per week (SD = 1.2) leading TT cohorts (see Table 7); those leading TT + cohorts spent 2.1 h (SD = 0.9). Supervisors leading TT cohorts spent 1.4 h per week *(SD* = 0.9), while those leading TT + cohorts spent 2.3 h (SD = 0.7).

Total therapist time per cohort for the 8-week program was 16.5 h (SD = 11.0) for TT and 18.4 h (*SD* = 7.80) for TT + . The 1.9-h increase for TT + (SE = 2.3) was not statistically significant (*p* = 0.42). Total supervisor time per cohort was 12.3 h (SD = 7.4 h) for TT and 19.9 h (SD = 6.3) for TT + . The 7.6-h increase for TT + (SE = 1.7), due in part to meeting with consultants, was statistically significant (*p* = 0.001, 95% CI 4.2–11.1-h increase). Finally, the 9.5 (SE = 4.8) hour increase in total hours for both therapists and supervisors in TT + was statistically significant (*p* = 0.05, 95% CI 0.05–19.0 h). Average weekly consultant time per cohort for TT + was 0.5 h and the total consultant time was 4 h.

**Table 7:** Average hours, average wages, and average costs

	Weekly hours		Total hours		Average hourly wages^a^	Cost	
Role	TT	TT +	TT	TT +		TT	TT +
Therapist	1.9 (1.2)	2.1 (0.9)	16.5 (11.0)	18.4 (7.9)	$34.50	$573 (379)	$632 (277)
Supervisor	1.4 (0.9)	2.3 (0.7)	12.3 (7.4)	19.9 (6.3)	$35.00	$429 (258)	$694 (218)
Total	3.3 (1.8)	4.4 (1.0)	28.8 (16.1)	38.3 (10.55)	$34.75	$1002 (561)	$1431^b^ (325)

^a^Costs were calculated at the level of the individual therapist and supervisor using each’s own hours and each’s own wage (which was based on the experience of each therapist and supervisor). They were not calculated by multiplying the average total hours times the average hourly wage

^b^Total TT + costs include clinical consultant cost

Translating hours into costs, the sum of the average total costs across therapists, supervisors, and consultants (the latter for TT +) was $1,002 (SD = 561) for TT and $1431 (SD = 325) for TT + (see Table 7). The difference was $429 (*SE* = 169), which was statistically significant (*p* = 0.01, 95% CI 98–760). Among the 109 students, the average improvement in MASC-2 scores for TT + was 1.69 points less (i.e., worse) than the average improvement for TT, which was not statistically significant (SE = 1.94, 95% CI − 2.11–5.49). The correlation of the difference in cost and fidelity score (used for calculating measures of sampling uncertainty for the cost-effectiveness outcomes) was − 0.072.

**Fig. 2:**
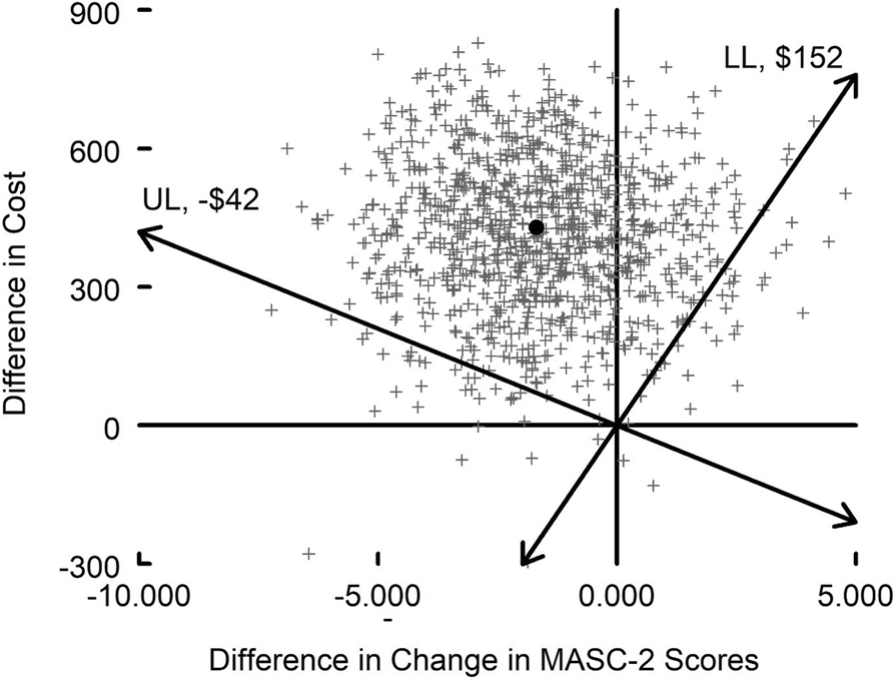
Cost-effectiveness plane. The point estimate (solid black circle) indicates TT + costs more and is less effective than TT

Figure 2 shows the distribution of the differences in cost and effect on the cost-effectiveness plane. The point estimates for the difference in cost and effect (solid black circle) indicate that TT + increased costs by $429 and decreased the improvement in MASC-2 scores by 1.69 points (i.e., reduced costs and yielded a greater movement in MASC-2 scores). A majority of the points fall in the quadrant indicating that TT dominates (i.e., costs less and is more effective) TT + , although there is an insufficient density for us to be 95% confident of dominance. The 95% confidence interval for the cost-effectiveness ratio ranges from a lower limit of $152 to an upper limit of − $42. This interval indicates that *if* our willingness to pay for a 1-point increase in the MASC-2 score is less than $152, we can be 95% confident that TT + is not a good value (i.e., that TT is a good value). If our willingness to pay is greater than $152, we cannot be 95% confident that the value of the two implementation strategies differs.

**Fig. 3:**
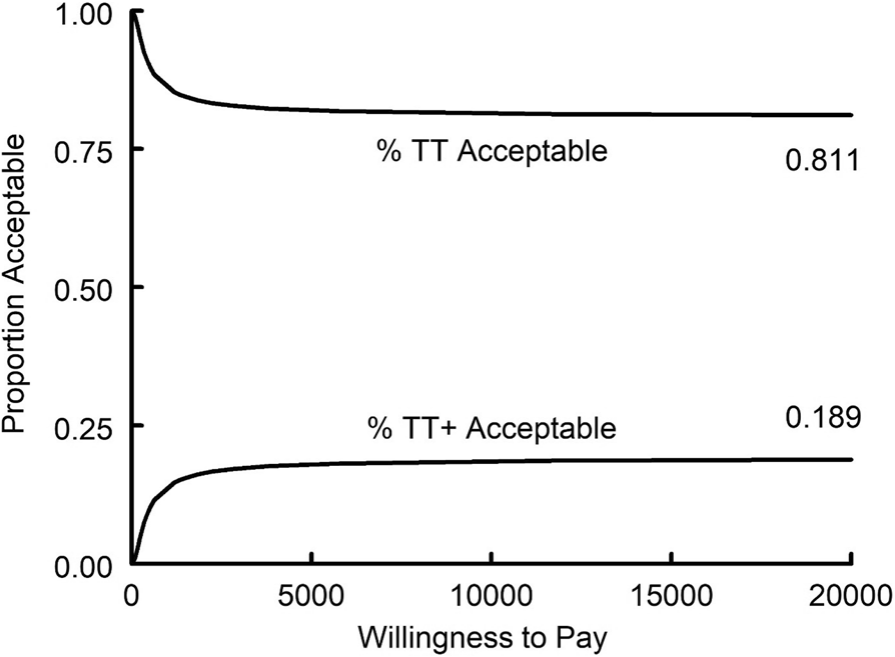
Cost-effectiveness acceptability curve. TT has at least an 81% chance of being cost-effective for all values of willingness to pay between $0 and $20,000 per one-point improvement in MASC-2 anxiety scores

The acceptability curve (Fig. 3), indicates that compared to TT + , TT has at least an 81% chance of being a good value for all values of willingness to pay between $0 and $20,000 for a 1-point improvement in MASC-2.

### Post hoc power estimation

With 10 schools (i.e., clusters) in TT and 12 schools in TT + , the MASC-2 rating scale effect size was estimated to be 0.2 SD, assuming a mean (SD) pre- to post-change score in the MASC-2 rating scale of TT = 3.26 (9.9), and TT +  = 1.27 (8.2). With the number of students in TT = 43 and in TT +  = 82, the power to detect an effect size = 0.2 SD was estimated to be 7%, which indicates that the results related to MASC 2 were unpowered to find condition by time interaction effects.

The power calculation for fidelity (implementation outcomes) is based on the reduced number of therapists from 60 to 37 therapists (TT = 16, TT +  = 21 therapists). We had a 23% power to detect a 5% difference in content fidelity between the two conditions.

## Discussion

The aims of the present study were to compare implementation outcomes, student outcomes, cost, and cost-effectiveness of two TT implementation strategies. In doing so, we advance the literature about the optimal design of TT strategies for under-resourced urban public schools and other similar resource-constrained settings.

Despite a higher level of support (i.e., external expert consultation post initial training) provided to supervisors in TT + , therapists in this condition showed similar levels of content and process fidelity, and a similar dosage of the treatment was provided in both conditions. Similar fidelity outcomes between these conditions may be due to relative similarities in the amount and type of support provided to therapists in each condition. Therapists in TT and TT + received the same amount of support during the initial training (i.e., training workshop) but also after initial training (i.e., supervision). The only difference between the conditions was the support provided to supervisors after the initial training (i.e., consultation). We were not able to find other studies testing therapist fidelity differences between two TT strategies to compare to results of the present study.

Therapists in both conditions showed high levels of content fidelity and moderate levels of process fidelity. Although prior implementation studies have used different measures and methods for measuring therapist fidelity (which could account for differences between the studies), the levels of content fidelity obtained in the present study are much higher compared to those obtained in studies employing train-the-trainer with community clinicians using an individual therapy approach with children with anxiety, depression, trauma or conduct problems [12], and by licensed outpatient and residential addiction and mental health clinicians conducting motivational interviewing [8]. The high levels of content fidelity and moderate levels of process fidelity in the present study could be the result of ongoing supervision provided to therapists by agency supervisors [4, 5].

Dosage was relatively low (an average of 6.32 sessions for TT, and 5.76 sessions for TT + attended out of an 8-session protocol) for both conditions. This was likely the result of time constraints in the school setting. Over the course of the academic year, students were not able to participate in group sessions because of state school-wide testing, school closures, and class trips. As a result, therapists often had to combine the content of two sessions into one session. Also, some treatment sessions were truncated at the end of the academic year or because schools closed due to COVID-19 during the 2019–2020 school year.

With regard to student outcomes, students who participated in groups conducted by therapists in the TT + and TT showed a similar small decrease across time in symptoms of anxiety (MASC-2 =  − 1.99 [1.56]) and depression (CDI-2 = 1.15 [1.39], and similar small improvement in academic engagement (EvsD Emotional Disaffection = 0.15 [0.57]). This finding might suggest that after a certain level of support is provided, a training approach based on “more is always better,” does not apply to the training of trainers. Indeed, the finding might have revealed a training threshold effect [16], in which providing support to supervisors beyond a certain level does not lead to significantly better outcomes. These results are consistent with findings from a previous study using individual therapy for the treatment of depression and anxiety [12]. Additionally, students across conditions showed increases in teacher-reported academic engagement on one subscale, emotional disaffection. The emotional disaffection subscale taps into students’ emotions indicating motivated withdrawal or alienation during learning activities [43]. Improvement in this scale indicates less enervated emotion (tired, sad, bored), alienated emotion (frustration, anger), and pressured participation (anxiety) [43]. No time effects were found for the other three subscales.

Given that student participants were recruited based on their status of being at-risk for an anxiety disorder, as opposed to their diagnostic status, their baseline scores tended to be below the clinical threshold and closer to the mean of the MASC-2 and CDI-2 rating scales norm samples. As a result, the post-treatment scores, while an improvement compared to baseline scores, had little to no room to show a larger improvement because the baseline scores were already low. The results are consistent with the literature showing smaller effect sizes for targeted group interventions implemented at school, compared to indicated individual interventions [45].

The results of the cost-effectiveness analyses indicated that therapists in both conditions spent, on average, the same amount of time preparing for and delivering group content to students. However, supervisors in the TT + condition spent more time supporting therapists in supervision and receiving consultation from consultants. Unlike supervisors in TT condition, supervisors in the TT + condition had to devote extra time to receiving a consultation. The sum of the average costs for therapists, supervisors, and consultants was significantly higher for TT + (i.e., $1431) than for TT (i.e., $1002). Thus, TT + increased costs without an improvement in MASC-2 scores. There was a greater than 80% chance that TT was a good value compared to TT + for all values of willingness to pay.

To the extent that weak evidence supports one implementation strategy over the other, TT is preferred to TT + . That is because there is an 80 + percent chance that TT is a good value compared to TT + for values of willingness to pay per one point improvement in anxiety scores from $0 to $20,000. If the willingness to pay is $152 per point or less, we can be 95% confident that TT is a good value. Mental health agencies that provide services in low-income urban schools, which rely almost exclusively on Medicaid funds to sustain their operations [46], may be less likely to want to risk spending more for no benefit. These agencies are increasingly expected to use EBPs with students [47] but are rarely compensated for indirect service activities such as supervision [48]. Identifying affordable approaches for supervision is key to meeting funder expectations [2, 49].

## Limitations

The results of the study should be considered in light of several study limitations. First, due to the COVID-19 pandemic, school closures in 2020 affected our ability to recruit new participants, deliver training, and collect post-intervention data. Ten groups were halted permanently. The resultant sample was smaller than expected for the group comparisons for implementation and effectiveness outcomes. For example, we only had a 7% power to detect an effect size of 0.2 SD, indicating that the results related to the MASC 2 anxiety scores were unpowered to find significant differences for the condition by time effects. We had 23% power to detect a 5-point difference in content fidelity between TT and TT + . Second, when the start of groups was delayed, supervisors in both conditions received a second initial training session within a month prior to the first treatment session. This likely made the level of support provided to therapists more similar than initially planned. Third, the study did not have a supervision-as-usual condition, which could be compared to each of the TT strategies. Therefore, it is unclear how the TT strategies compare to clinical services provided to students by therapists under varying levels of supervision. Fourth, the study did not collect data on the sustainment of the implementation strategies. Future studies would examine whether the potential benefits of an enhanced train-the-trainer strategy over a regular train-the-trainer strategy would emerge during a sustainment period.

## Conclusion

The results of the study suggest that the TT implementation approach, which provided mental health agency therapists with a thorough training workshop on how to be effective supervisors, as well as a thorough training workshop and ongoing supervision to therapists on how to implement EBPs, is sufficient for achieving adequate levels of therapist fidelity and child outcomes. This approach seems to have the additional benefit of having a significantly lower cost than TT + . This study advances the implementation science literature by demonstrating the amount and type of support provided within a TT implementation strategy that leads to acceptable levels of fidelity, at a reasonable cost.

### Supplementary Information

**Additional file 1:** **Supplementary Information 1.** Training of clinical supervisors, therapists, and research consultants.

## Data Availability

De-identified data used in the study can be obtained upon request from the first author.

## Abbreviations

BD: Behavioral disaffection
BE: Behavioral engagement
CBT: Cognitive Behavioral Therapy
CDI-2: Children’s Depression Inventory–2nd Edition
CATS: CBT for Anxiety Treatment in Schools
DS: Delivery System
ED: Emotional disaffection
EE: Emotional engagement
EFA: Explanatory factor analysis
EvsD: Engagement versus Disaffection with Learning
ICC: Intraclass correlation
ISF: Interactive Systems Framework
ITT: Intention to treat
KT: Knowledge Test
MASC-2: Multidimensional Anxiety Scale for Children-2nd Edition
PCIT: Parent-Child Interaction Therapy
REDCap: Research Electronic Data Capture
SCARED: Screen for Child Anxiety-Related Disorders
SD: Standard deviation
SE: Standard error
SS: Support System
STS: Synthesis and Translation System
TT: Train-the-Trainer
TT +: Train-the-Trainer plus
