# Supplementary material for: Implementation fidelity, student outcomes, and cost-effectiveness of train-the-trainer strategies for Masters-level therapists in urban schools: results from a cluster randomized trial
Source: Implement Sci. 2024 Jan 25;19:4. doi: 10.1186/s13012-023-01333-9 (PMC10809609; doi:10.1186/s13012-023-01333-9)
Supplement: Supplementary file 1 — Additional file 1: Supplementary Information 1. Training of clinical supervisors, therapists, and research consultants. [file 13012_2023_1333_MOESM1_ESM.docx]

**Supplementary Information 1: Training of clinical supervisors, therapists, and research consultants**

The training and support procedures for supervisors and therapists in this proposal were informed by the Support System and Delivery System of the Interactive Systems Framework (ISF) [1], implementation studies in nontraditional settings [2-4], a review of training studies conducted by a member of our team [5], and a randomized clinical trial of different types of support for clinicians [6].

**ISF SUPPORT SYSTEM - General capacity building –** The study was conducted in the context of capacity building, specifically, training and consultation, to enable agency supervisors to become effective clinical supervisors. Research consultants conducted approximately 20 hours of initial training in August of each year for all agency supervisors and therapists. The training content included a competency framework for supervisors [7], strategies for identifying children who could benefit from the service (e.g., conducting in-service presentations to school faculty on the signs of anxiety in children), and how to access Zoom and REDCap while in the field (e.g., wireless access; using fidelity forms). They were also trained on the use of fidelity monitoring and conducting performance feedback with therapists. All supervisors received a supervision manual and a procedures manual.

**Innovation-specific capacity building –** Supervisors learned how to prepare therapists for each treatment session and how to measure treatment fidelity and conduct performance feedback using fidelity forms. Therapists were trained in using a screening instrument (SCARED) [8] for the identification of potential participants. Therapists were introduced to a competency model for CBT [9] which delineates generic therapeutic competencies (e.g., professional practice), CBT competencies (e.g., relevance of theory and research) and specific CBT techniques (e.g., managing negative thoughts). They also learned about how to deal with implementation barriers (e.g., scheduling sessions, conducting exposure tasks) [10]. Research consultants followed training procedures used in other EBP dissemination studies in nontraditional settings [2, 11] and other training strategies that have been found to be effective (i.e., active learning such as modeling and role-playing)

After the initial training workshop, research consultants provided 10 weekly 60-minute consultation sessions to supervisors in TT+. The consultation was condensed into 5 sessions for returning supervisors in subsequent project years. The consultation focused on implementation of activities in the treatment manual for upcoming sessions. Consultants primarily employed didactic, discussion-based strategies plus the use of coaching, including encouraging supervisors to self-reflect on previous sessions (what went right, what went wrong), video-based performance feedback, and role-playing to improve performance. All schools were also provided with a DVD including sample session content, and they were encouraged to watch the session examples at their leisure.

**ISF DELIVERY SYSTEM - General capacity use.** When the start of the group was delayed past a certain time after the initial training, supervisors in both conditions received a second initial training session within a month prior to the first treatment session. The purpose of the second session was to review key points from the training (e.g., conducting performance feedback) and to answer questions about any aspect of the supervision process. Supervisors were expected to conduct supervision with therapists. Therapists were expected to conduct CATS groups with fidelity. However, we did not enforce these expectations so as to not introduce confounds to the data.

**Innovation-specific capacity use.** Supervisors provided one 50-minute supervision session for each treatment session the therapist conducted. The session was divided into two distinct categories: (a) group preparation (i.e., discussing referrals, preparing for upcoming session, engaging in problem solving around implementation barriers), and (b) coaching (e.g., performance feedback).

*Group Preparation.* The main goal of this portion of supervision was to teach upcoming session content. Supervisors were to review content in the treatment manual (e.g., main session content; active learning activities) in order to prepare the therapist to conduct the upcoming session.

*Coaching.* The goal of this portion of supervision was to provide performance-based feedback on the previous treatment session. Prior to the session, the supervisor logged onto REDCap and ShareFile, watched a recording of the previous session, completed the fidelity data form, and identified clips for discussion using estimated timestamps. The supervisor engaged in a discussion of the therapist’s self-reflection. Then, the supervisor provided the therapists with fidelity data and showed video clips from the previous session regarding effective and ineffective implementation of CATS. Supervisors provided therapists fidelity data with regard to *content* (i.e., the material they were supposed to cover in session), and *process* (i.e., how well they delivered the session). During these meetings, the therapists’ goal was to reach a high level of content and process fidelity (set at > 80% on the CFC and process fidelity set at an overall score > 3 on the PFC). All session video recordings, and fidelity levels per session were available to therapists for on-demand examination for the duration of their participation in the study (i.e., one year).

**Compensation.** Supervisors were compensated for each provided supervision session with therapists and for attending each consultation session with research consultants. Therapists were compensated for each supervision session attended with their supervisor. The amount of compensation was based on projected time commitment to the project. Parents and teachers received a small stipend and children received small gifts upon completing study measures.

**References**

1. Wandersman A, Duffy J, Flashpohler P, Noonan R, Lubell K, Stillman L, et al. Bridging the gap between prevention research and practice: The Interactive Systems Framework for dissemination and implementation. American journal of community psychology. 2008;41(3-4):171-81.
2. Lochman JE, Boxmeyer C, Powell N, Qu L, Wells K, Windle M. Dissemination of the Coping Power program: importance of intensity of counselor training. J Consult Clin Psychol. 2009 Jun;77(3):397-409.
3. Bickman L, Kelley SD, Breda C, de Andrade AR, Riemer M. Effects of routine feedback to clinicians on mental health outcomes of youths: results of a randomized trial. Psychiatric services. 2011 Dec;62(12):1423-9.
4. Schoenwald SK, Mehta TG, Frazier SL, Shernoff ES. Clinical Supervision in Effectiveness and Implementation Research. 2013;20:44-59.
5. Beidas RS, Kendall PC. Training Therapists in Evidence-Based Practice: A Critical Review of Studies From a Systems-Contextual Perspective. Clinical Psychology Science and Practice. 2010 Mar;17(1):1-30.
6. Sholomskas DE, Syracuse-Siewert G, Rounsaville BJ, Ball SA, Nuro KF, Carroll KM. We don't train in vain: a dissemination trial of three strategies of training clinicians in cognitive-behavioral therapy. J Consult Clin Psychol. 2005 Feb;73(1):106-15.
7. American Psychological Association (APA). Guidelines for clinical supervision in health service psychology. American Psychologist. 2015;70(1):33-46.
8. Birmaher B, Brent DA, Chiappetta L, Bridge J, Monga S, Baugher M. Psychometric properties of the Screen for Child Anxiety Related Emotional Disorders (SCARED): a replication study. J Am Acad Child Adolesc Psychiatry. 1999 Oct;38(10):1230-6.
9. Sburlati ES, Schniering CA, Lyneham HJ, Rapee RM. A Model of Therapist Competencies for the Empirically Supported Cognitive Behavioral Treatment of Child and Adolescent Anxiety and Depressive Disorders. Clinical child and family psychology review. 2011;14(1).
10. Beidas RS, Mychailyszyn MP, Edmunds JM, Khanna MS, Downey MD, Kendall PC. Training school mental health providers to deliver cognitive-behavioral therapy. School Mental Health. 2012;4:197-206.
11. Martin JL, Weisz JR, Chorpita BF, Higa CK, Wells KC, Southam-Gerow MA. Moving evidence-based practices into everyday clinical care settings: Addressing challenges associated with pathways to treatment, child characteristics, and structure of treatment. Emotional & Behavioral Disorders in Youth. 2007;7:5-21.
